# Angiogenic and Inflammatory Markers of Cardiopulmonary Changes in Children and Adolescents with Sickle Cell Disease

**DOI:** 10.1371/journal.pone.0007956

**Published:** 2009-11-23

**Authors:** Xiaomei Niu, Mehdi Nouraie, Andrew Campbell, Sohail Rana, Caterina P. Minniti, Craig Sable, Deepika Darbari, Niti Dham, N. Scott Reading, Josef T. Prchal, Gregory J. Kato, Mark T. Gladwin, Oswaldo L. Castro, Victor R. Gordeuk

**Affiliations:** 1 Center for Sickle Cell Disease, Howard University, Washington, D. C., United States of America; 2 Department of Hematology, Children's National Medical Center, Washington, D. C., United States of America; 3 Department of Cardiology, Children's National Medical Center, Washington, D. C., United States of America; 4 Department of Pediatrics, University of Michigan, Ann Arbor, Michigan, United States of America; 5 Pulmonary and Vascular Medicine Branch, National Heart, Lung and Blood Institute, Clinical Center, National Institutes of Health, Bethesda, Maryland, United States of America; 6 University of Utah, ARUP Institute of Clinical and Experimental Pathology, and Veterans Administration Hospital, Salt Lake City, Utah, United States of America; 7 Division of Pulmonary, Allergy and Critical Care Medicine, University of Pittsburgh Medical Center and Hemostasis and Vascular Biology Research Institute, University of Pittsburgh, Pittsburgh, Pennsylvania, United States of America; New York University School of Medicine, United States of America

## Abstract

**Background:**

Pulmonary hypertension and left ventricular diastolic dysfunction are complications of sickle cell disease. Pulmonary hypertension is associated with hemolysis and hypoxia, but other unidentified factors are likely involved in pathogenesis as well.

**Design and Methods:**

Plasma concentrations of three angiogenic markers (fibroblast growth factor, platelet derived growth factor–BB [PDGF-BB], vascular endothelial growth factor [VEGF]) and seven inflammatory markers implicated in pulmonary hypertension in other settings were determined by Bio-Plex suspension array in 237 children and adolescents with sickle cell disease at steady state and 43 controls. Tricuspid regurgitation velocity (which reflects systolic pulmonary artery pressure), mitral valve E/Edti ratio (which reflects left ventricular diastolic dysfunction), and a hemolytic component derived from four markers of hemolysis and hemoglobin oxygen saturation were also determined.

**Results:**

Plasma concentrations of interleukin-8, interleukin-10 and VEGF were elevated in the patients with sickle cell disease compared to controls (P≤0.003). By logistic regression, greater values for PDGF-BB (P = 0.009), interleukin-6 (P = 0.019) and the hemolytic component (P = 0.026) were independently associated with increased odds of elevated tricuspid regurgitation velocity while higher VEGF concentrations were associated with decreased odds (P = 0.005) among the patients with sickle cell disease. These findings, which are consistent with reports that PDGF-BB stimulates and VEGF inhibits vascular smooth muscle cell proliferation, did not apply to E/Etdi.

**Conclusions:**

Circulating concentrations of angiogenic and pro-Inflammatory markers are altered in sickle cell disease children and adolescents with elevated tricuspid regurgitation velocity, a subgroup that may be at risk for developing worsening pulmonary hypertension. Further studies to understand the molecular changes in these children are indicated.

## Introduction

Pulmonary hypertension and left ventricular diastolic dysfunction are found in up to 32% and 18% of adults with sickle cell disease, respectively, and are associated with reduced exercise tolerance and high mortality [1], [2], [3]. Elevated estimated systolic pulmonary artery pressures [4], [5], [6] and left ventricular stiffness [7] are recognized to develop in children with sickle cell disease but the clinical importance is not clear [7], [8]. Echocardiography can be employed to non-invasively estimate systolic pulmonary artery pressure and left ventricular diastolic function. Tricuspid regurgitation velocity reflects systolic pulmonary artery pressure [1] and the ratio of early diastolic mitral inflow velocity to early diastolic tissue Doppler imaging annular velocity (E/Etdi) reflects left ventricular diastolic function [9], [10].

The Pulmonary Hypertension and Hypoxic Response in Sickle Cell Disease (PUSH) Study is an on-going multicenter observational study that was designed to identify factors in pre-symptomatic children with sickle cell disease that are predictors of cardiopulmonary complications and that therefore are potential mechanistic targets for early preventive intervention. Using a conservative definition of ≥2.6 m/sec, we recently reported that 11% of 310 children and adolescents with sickle cell disease had elevated tricuspid regurgitation velocity at steady state and that elevated velocity had independent associations with degree of hemolysis and with lower hemoglobin oxygen saturation [6]. We also reported that 5% had elevated E/Etdi [6]. Although a functional deficit was not found in the children with elevated tricuspid regurgitation velocity as measured by the six-minute walk test, there was a statistically significant decrease in oxygenation during the test. This observation indicates that the children with elevation of tricuspid regurgitation velocity are biologically different with respect to pulmonary vascular function compared to those with lower velocities. Thus, albeit pending the results of long term studies, elevated tricuspid regurgitation velocity may be an early marker of disease that identifies a subset of sickle cell disease patients at risk for developing pulmonary hypertension later in life and that could be used to discover the early underlying mechanisms of pulmonary hypertension.

The factors that lead to the development of cardiopulmonary complications in the setting of sickle cell disease have not been fully identified. Studies in both adults [1] and children [5], [6] with sickle cell disease have found correlations between hemolysis and pulmonary hypertension. Intravascular hemolysis contributes to a hemolytic vasculopathy in part through scavenging nitric oxide, a key modulator of microvascular function [11], and through limiting availability of arginine, the substrate for nitric oxide synthase [12]. Hemolysis does not fully explain the finding of pulmonary hypertension in this setting, for pulmonary hypertension develops in patients with hemoglobin SC or Sβ+-thalassemia, conditions with markedly less hemolysis than hemoglobin SS [1], [13]. In addition and possibly related to the hemolytic process, hypoxia may be a contributing factor [6]. Humans exposed to chronic hypoxia have a tendency to develop pulmonary hypertension [14], and patients with sickle cell disease may experience hypoxia due to anemia, chronic hemoglobin oxygen desaturation, upper airway obstruction and repeated episodes of vasoocclusive pain crisis and/or acute chest syndrome [15], [16], [17], [18], [19], [20]. Hypercoagulability [21], platelet activation [22] and up-regulation of the inflammatory response [21] have also been proposed as contributing to the development of pulmonary hypertension in sickle cell disease. Although a number of biological pathways have been implicated in sickle cell disease, few molecular targets have been identified.

Altered expression of inflammatory molecules and angiogenic growth factors have been observed in primary pulmonary hypertension and/or non-sickle cell disease-related forms of secondary pulmonary hypertension. These markers include interleukin-6 [23], [24], interleukin-8 [25], interleukin-10 [26], tumor necrosis factor-α [27], monocyte chemoattractant protein-1 [25], RANTES [28], vascular endothelial growth factor (VEGF) [29], platelet-derived growth factor (PDGF) [30] and fibroblast growth factor [31]. Likewise, increased exposure to inflammatory cytokines such as interleukin-6 and tumor necrosis factor-α [32], [33] and angiogenic factors such as PDGF and VEGF [34], [35] has been associated with altered cardiac function in settings other than sickle cell disease. Our hypothesis is that inflammatory and angiogenic factors contribute to the pathogenesis of cardiopulmonary complications in sickle cell disease. We therefore measured circulating concentrations of inflammatory and angiogenic markers and correlated them with cardiopulmonary findings in a subgroup of children and adolescents with sickle cell disease enrolled in the PUSH Study.

## Methods

### Study Participants

This report includes a subset of children and adolescents with sickle cell disease from 3 to 20 years of age and control participants who were evaluated at steady state as previously described [6]. Controls were matched by age, sex and ethnicity to every sixth patient with sickle cell disease enrolled in the study and included individuals with hemoglobin AA, hemoglobin AS or hemoglobin AC. This report includes 237 patients and 43 controls who were enrolled in the study as of early 2008. Patients had hemoglobin SS, SC, Sβ-thalassemia or other major sickling phenotypes as determined by hemoglobin electrophoresis or HPLC and confirmed by molecular genetic testing (see below). Hemoglobins S and C are mutated forms of hemoglobin while hemoglobin A is the normal form. β-thalassemia refers to mutations in which there is reduced or absent production of β-globin. Two copies of mutated forms indicate hemolytic anemia such as hemoglobin SS disease (sickle cell anemia), hemoglobin SC disease or sickle-β-thalassemia. Patients had to be at least three weeks out from hospitalization or emergency room visit for acute pain, vasoocclusion or infection and controls had to be at least three weeks out from acute infections or other illnesses. Participants were recruited at three centers: Howard University and Children's National Medical Center in Washington, DC and the University of Michigan in Ann Arbor, Michigan. The research was approved by the IRBs of each participating institution and written consent was obtained for all participants. Hemoglobin oxygen saturation was measured by pulse oximetry. Reticulocyte count, lactate dehydrogenase, aspartate aminotransaminase and total bilirubin were analyzed by automated methodology at each institution and used to develop the hemolytic component described in the Statistical analysis section below. Among the patients with sickle cell disease 11 (4.7%) had mitral valve E/Etdi ≥9.22, 46 (21.1%) had tricuspid regurgitation velocity ≥2.5 m/sec, and 21 (9.6%) had regurgitation velocity ≥2.6 m/sec. Tricuspid regurgitation velocity elevations of this magnitude predict only mild elevations in systolic pulmonary artery pressure and their functional significance has not been determined in children and adolescents with sickle cell disease. Among adults with sickle cell disease, such elevations are associated with high mortality during two years of follow-up [1].

### Molecular Genetic Testing

Hemoglobin S, C genotyping was conducted at ARUP Laboratories (Salt Lake City, UT) using loci-spanning probe – PCR as described[36]. Genotyping was performed using the LightCycler480 (LC480) Real-Time PCR System (Roche Applied Science, Indianapolis, IN) using 100 ng DNA, 5 µL LightCycler 480 Probes Master (2x) mix (Roche Applied Sciences), 0.2 nM PCR primers, 0.5 nM LSProbes (Idaho Technology, Salt Lake City, UT) in a 10 µL PCR reaction. Cycling parameters were modified from those previously described for the LC480 System. The LC480 parameters were: denaturation at 94°C for 10 minutes, followed by 40 cycles of 95°C for 10 seconds, 63°C for 1 minute, and 75°C for 5 seconds. Loci-spanning probe melt analysis was preformed by continuous signal acquisition between 38°C – 65°C at default ramp rate of 0.6°C/s and 5 acquisitions per °C.

### Analysis of Biological Markers at Baseline

Plasma concentrations of ten biological markers were assayed in a subgroup of patients and controls using the Bio-Plex suspension array system (Bio-Rad, Hercules, CA): interleukins-6, 8 and 10, interferon-γ, tumor necrosis factor-α, monocyte chemoattractant protein-1, basic fibroblast growth factor, PDGF (platelet-derived growth factor)-BB, VEGF (vascular endothelial growth factor) and RANTES (Regulated upon Activation, Normal T-cell Expressed, and Secreted). The system allows simultaneous identification of cytokines in a 96 well filter plate. In brief, the appropriate cytokine standards and samples diluted in plasma diluents were added to a 96 well filter plate. The samples were incubated at room temperature for 30 minutes with antibodies chemically attached to fluorescent-labeled micro beads. After three filter washes, premixed detection antibodies were added to each well and incubated for 30 min. Following three washes, premixed streptavidin-phycoerythrin was added to each well and incubated for 10 minutes followed by three more washes. Then beads were re-suspended with 125 µl of assay buffer and the reaction mixture was quantified using the Bio-Plex protein array reader. Data were automatically processed and analyzed by Bio-Plex Manager Software 4.1 using the standard curve produced from recombinant cytokine standard.

### Echocardiographic Measurements of Cardiopulmonary Status

Doppler echocardiography was employed to estimate systolic pulmonary artery pressure through measurement of the tricuspid regurgitation velocity and to evaluate left ventricular diastolic function by determining the mitral valve E/Etdi ratio as previously described [6]. Transthoracic echocardiography was performed using the Philips Sono 5500/7500 or iE33, Acuson Sequoia, or General Electric VIVID 7 or VIVID I instruments. Cardiac images were obtained, measurements performed, and studies interpreted centrally according to guidelines of the American Society of Echocardiography. The tricuspid regurgitation velocity was measurable in 92% of the patients studied, and patients with unmeasurable velocity were excluded from analyses that involved the velocity. Based on the mean +2SD in controls, an elevated tricuspid regurgitation velocity of ≥2.60 m/sec and a mitral valve E/Etdi ratio of ≥9.22 were considered to be elevated.

### Statistical Analysis

For continuous variables that did not follow a normal distribution, the best transformation to a normal distribution was made for statistical analyses. Principal component was used to derive a hemolytic component from reticulocyte count, lactate dehydrogenase, aspartate transaminase and bilirubin. Principal component analysis produces a number of components equal to the number of variables in the analysis; each component represents a normalized standard distribution with a mean value of 0. Continuous variables were compared between two groups with the student t-test or with analysis of variance. Relationships among continuous variables were determined by bivariate linear regression. Independent associations of tricuspid regurgitation velocity of 2.60 m/sec or higher and mitral valve E/Etdi of 9.22 or higher with hemolytic component, hemoglobin oxygen saturation and biologic markers were assessed by logistic regression. The most parsimonious models were developed that included only variables with P<0.05 in the final model. Models were checked for model assumption and fitness. Analyses were performed with STATA 10 (StataCorp, College Station, TX). AMOS 17.0 software (SPSS, Chicago, IL) was used to develop a mechanistic pathway analysis model of tricuspid regurgitation velocity based on factors that were significant, independent predictors of elevated tricuspid regurgitation velocity by logistic regression. The final pathway model included only parameters and relationships that were statistically significant (P<0.05). The effect of each relationship was presented with a standardized coefficient.

## Results

### Biologic Markers in Sickle Cell Disease Patients and Controls

The median age of the 237 patients with sickle cell disease was 12 years and 46% were males. The median age of the 43 controls was 13 years and 51% were males. Seventy-five percent of the patients had the severe sickling genotypes of hemoglobin SS (n = 174), hemoglobin Sβ^0^-thalassemia (n = 3) or hemoglobin SD^LA^ (n = 1), 38% were receiving hydroxyurea and 13% were on a chronic transfusion program. The median tricuspid regurgitation velocity was 2.3 m/sec in patients compared to 2.1 m/sec in controls (P = 0.0004 by the student t-test). The median mitral valve E/Etdi ratio was 6.4 in patients compared to 6.3 in controls (P = 0.5). After adjustment for hydroxyurea therapy by analysis of variance, patients with sickle cell disease had higher plasma interleukin-8, interleukin-10, and VEGF concentrations compared to the control participants and lower plasma RANTES concentrations (Table 1). Concentrations of tumor necrosis factor-α, interferon-γ, VEGF and fibroblast growth factor were significantly higher in patients with severe sickling phenotype than those with mild sickling phenotype (data not shown).

**Table 1:** Plasma concentrations of biologic markers in patients with sickle cell disease and control participants; results in median (interquartile range).

	Sickle cell disease patients (N = 237)	Controls (N = 43)	P^1^
**Inflammatory markers**			
Interleukin-6 (pg/ml)	0.8 (0.3–1.6)	0.4 (0.2–1.3)	0.031
Interleukin-8 (pg/ml)	0.4 (0.2–1.1)	0.3 (0.05–0.7)	0.001*
Interferon-γ (pg/ml)	40 (16–73)	34 (11–51)	0.2
Monocyte chemoattractant protein-1 (pg/ml)	7.0 (3.7–12.9)	8.1 (2.3–12.2)	0.4
Tumor necrosis factor-α (pg/ml)	25 (6–50)	10 (2–42)	0.1
Interleukin-10 (pg/ml)	1.6 (0.4–5.7)	1.1 (0.1–2.5)	0.003*
RANTES (ng/ml)	4.0 (2.4–5.8)	6.9 (3.5–1.5)	0.0002*
**Angiogenic markers**			
Fibroblast growth factor (basic) (pg/ml)	13.5 (3.3–32.5)	7.2 (5.1–17.0)	0.009
PDGF- BB (ng/ml)	0.4 (0.2–0.6)	0.3 (0.2–0.4)	0.043
VEGF (pg/ml)	1.6 (0.3–6.0)	0.5 (0.1–2.3)	0.002*

1 From ANOVA adjusted for hydroxyurea treatment and with best transformation of the variable.

* Statistically significant after Bonferroni adjustment for multiple comparisons.

### Relationships Among the Biologic Markers in the Patients with Sickle Cell Disease

Of the inflammatory markers, relationships were observed among interleukin-6, interleukin-8, interleukin-10, interferon-γ and tumor necrosis factor-α with Pearson's correlation coefficients of 0.55 to 0.75 (P<0.0001). Monocyte chemoattractant protein-1 associated with interleukin-6 and interferon-γ with correlation coefficients of 0.55 and 0.56. Similarly, relationships were observed among all three angiogenic markers, VEGF, PDGF-BB and basic fibroblast growth factor, with Pearson's correlation coefficients of 0.57 to 0.82. There were also relationships between the inflammatory markers and angiogenic markers. Interleukin-8 and interferon-γ associated with all three angiogenic markers with correlation coefficients of 0.50 to 0.73. Interleukin-6, interleukin-10 and tumor necrosis factor-α associated with VEGF and fibroblast growth factor with correlation coefficients of 0.58 to 0.78.

### Relationship of Biologic Markers to Hydroxyurea Therapy and Chronic Transfusion Program in Patients with Sickle Cell Disease

Plasma concentrations of PDGF-BB (medians of 0.3 ng/ml versus 0.4 ng/ml, P = 0.0007) but not the other markers were lower in 89 patients receiving hydroxyurea compared to 148 patients not receiving the medication. Plasma concentrations of PDGF (medians of 0.8 ng/ml versus 0.3 ng/ml, P<0.0001) were higher in 32 patients on a chronic transfusion program compared to 201 patients not receiving chronic transfusions.

### Relationship of Biologic Markers with the Hemolytic Component, Hemoglobin Oxygen Saturation and Hemoglobin Concentration in Patients with Sickle Cell Disease

We recently reported independent associations of higher hemolytic component and lower hemoglobin oxygen saturation with elevated tricuspid regurgitation velocity in this cohort of patients with sickle cell disease [6]. We assessed the relationships of the biologic markers measured in this study with the hemolytic component, with hemoglobin oxygen saturation, and with hemoglobin concentration. Plasma concentrations of interleukin-8 correlated directly with the hemolytic component. Plasma concentrations of interleukin-8 and fibroblast growth factor correlated inversely with hemoglobin oxygen saturation. Plasma concentrations of interferon-γ, tumor necrosis factor-α, fibroblast growth factor and VEGF correlated inversely with the hemoglobin concentration (Table 2).

**Table 2:** Bivariate relationships of biologic markers with hemolytic component and hemoglobin oxygen saturation in patients with sickle cell disease [Pearson correlation].

	Hemolytic component (N = 225)		Hemoglobin O_2_ saturation (N = 233)		Hemoglobin concentration (N = 237)	
	R	P	R	P	R	P
**Inflammatory markers**						
Interleukin-6 (pg/ml, natural log)	0.07	0.3	−0.10	0.1	−0.08	0.2
Interleukin-8 (pg/ml, natural log)	0.21	0.001*	−0.20	0.002*	−0.12	0.1
Interferon-γ (pg/ml, natural log)	0.15	0.021	−0.14	0.034	−0.22	0.0006*
Monocyte chemoattractant protein-1 (pg/ml, natural log)	−0.07	0.3	0.01	0.9	−0.05	0.4
Tumor necrosis factor-α (pg/ml, natural log)	0.17	0.012	−0.13	0.049	−0.19	0.004*
Interleukin-10 (pg/ml, natural log)	0.15	0.026	−0.18	0.006	−0.14	0.021
RANTES (ng/ml, natural log)	−0.03	0.7	0.04	0.6	0.17	0.007
**Angiogenic markers**						
Fibroblast growth factor (basic) (pg/ml, sq. root)	0.15	0.022	−0.19	0.004*	−0.24	0.0001*
PDGF- BB (ng/ml, square root)	0.15	0.028	−0.15	0.025	−0.08	0.2
VEGF (pg/ml, natural log)	0.17	0.012	−0.16	0.016	−0.19	0.003*

* Statistically significant after adjustment for multiple comparisons.

### Bivariate Analysis of the Relationship of Biologic Markers with Cardiopulmonary Findings in Participants with Sickle Cell Disease (Table 3)

Interleukin 6, interleukin 8, interferon-γ, tumor necrosis factor-α, RANTES and PDGF-BB correlated positively with tricuspid regurgitation velocity. Similar results were obtained when the patients were stratified according to whether or not they were being treated with hydroxyurea, although the associations were not always statistically significant in these sub-analyses (data not shown). None of biological markers correlated significantly with the E/Etdi ratio by bivariate analysis.

**Table 3:** Bivariate relationships of biologic markers with cardiopulmonary outcomes in patients with sickle cell disease.

	Tricuspid regurgitation velocity (N = 218)		Mitral valve E/Etdi (N = 233)	
	R	P	R	P
**Inflammatory markers**				
Interleukin-6 (pg/ml, natural log)	0.20	0.004*	0.01	0.9
Interleukin-8 (pg/ml, natural log)	0.20	0.003*	0.07	0.3
Interferon-γ (pg/ml, natural log)	0.24	<0.001*	−0.004	0.9
Monocyte chemoattractant protein-1 (pg/ml, natural log)	0.11	0.09	−0.01	0.9
Tumor necrosis factor-α (pg/ml, nat. log)	0.22	0.001*	0.05	0.4
Interleukin-10 (pg/ml, natural log)	0.15	0.032	0.03	0.6
RANTES (ng/ml, natural log)	0.21	0.002*	−0.04	0.6
**Angiogenic markers**				
Fibroblast growth factor (basic) (pg/ml, sq. root)	0.10	0.16	0.07	0.3
PDGF- BB (ng/ml, square root)	0.20	0.003*	0.09	0.2
VEGF (pg/ml,natural log)	0.12	0.08	0.09	0.2

* Statistically significant after adjustment for multiple comparisons.

### Independent Associations of Biologic Markers with Cardiopulmonary Findings by Logistic Regression Analyses in Participants with Sickle Cell Disease

There are potential relationships among the expression of angiogenic growth factors and the inflammatory response in sickle cell disease [37], and we observed relationships among and between the inflammatory and angiogenic markers in this study as described above. Furthermore, we observed associations of some of the biologic markers measured with the hemolytic index and/or the hemoglobin oxygen saturation (Table 2), which we previously showed to be associated with elevated tricuspid regurgitation velocity [6]. It was therefore important to do analyses to search for independent relationships of biologic markers with the cardiopulmonary findings of interest in this study.

#### Tricuspid regurgitation velocity

We used logistic regression to examine the independent relationships of biologic markers, hemolytic component and hemoglobin oxygen saturation with tricuspid regurgitation velocity dichotomized as ≥2.6 m/sec versus <2.6 m/sec (Table 4). Greater values for PDGF-BB (P = 0.009), interleukin-6 (P = 0.019) and the hemolytic component (P = 0.026) were each independently associated with increased odds of elevated tricuspid regurgitation velocity while higher VEGF concentrations were associated with decreased odds (P = 0.005). We performed separate analyses in patients with severe sickling phenotype and mild sickling phenotype, and observed similar results to the overall analysis in both subgroups. However, the relationships in the mild sickling phenotype did not reach statistical significance because of the small sample size.

**Table 4:** Independent predictors of tricuspid regurgitation velocity ≥2.60 m/sec by logistic regression.

	Odds ratio (95% confidence interval)	P
VEGF (pg/ml, natural log)	0.60 (0.42–0.86)	0.005
PDGF-BB (ng/ml, square root)	10.0 (1.77–56.29)	0.009
Interleukin-6 (pg/ml, natural log)	1.60 (1.08–2.38)	0.019
Hemolytic component (relative unit)	1.44 (1.05–1.98)	0.026

186 patients with tricuspid regurgitation velocity <2.6 m/sec; 20 with velocity ≥2.6 m/sec. Variables entered into the analyses hemolytic component, hemoglobin oxygen saturation, chronic transfusion program, hydroxyurea therapy, interleukin-6, interleukin-8, tumor necrosis factor-α, interferon-γ, interleukin-10, RANTES, basic fibroblast growth factor, PDGF-BB and VEGF.

Hemoglobin oxygen saturation did not have a significant independent association with tricuspid regurgitation velocity after adjustment for the hemolytic component, VEGF, PDGF and interleukin-6 in the logistic regression model. Hydroxyurea therapy and chronic transfusion program did not have significant associations either.

#### Mitral valve E/Etdi ratio

None of the biologic markers had a significant association with E/Etdi ratio of 9.22 or higher versus less than 9.22.

### Complex Relationship of VEGF with Other Biologic Markers and with Tricuspid Regurgitation Velocity

**Figure 1:**
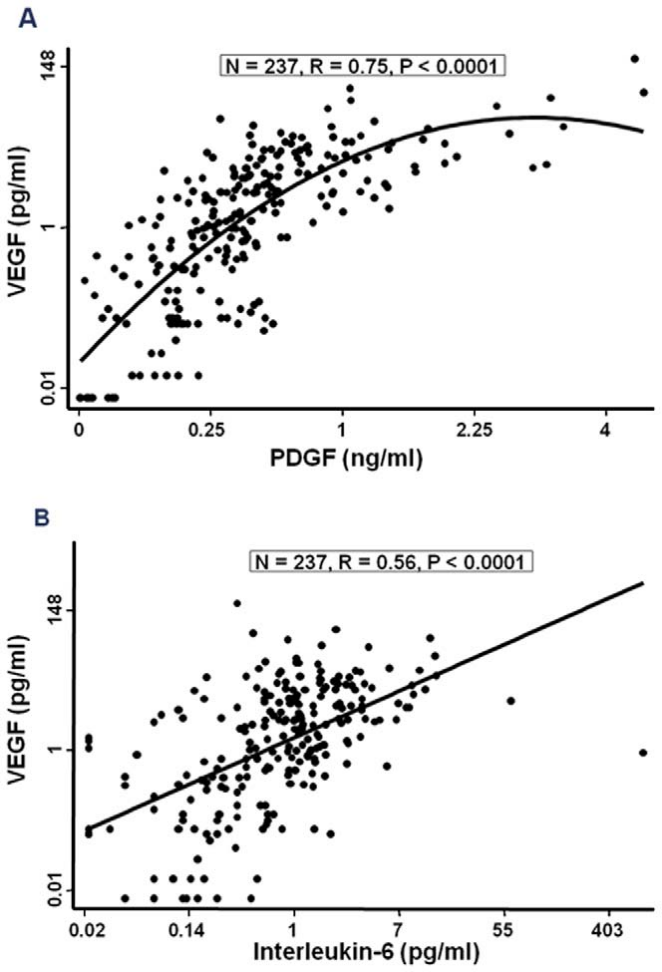
Bivariate relationships of VEGF with PDGF-BB and interleukin-6 in patients with sickle cell disease.

**Figure 2:**
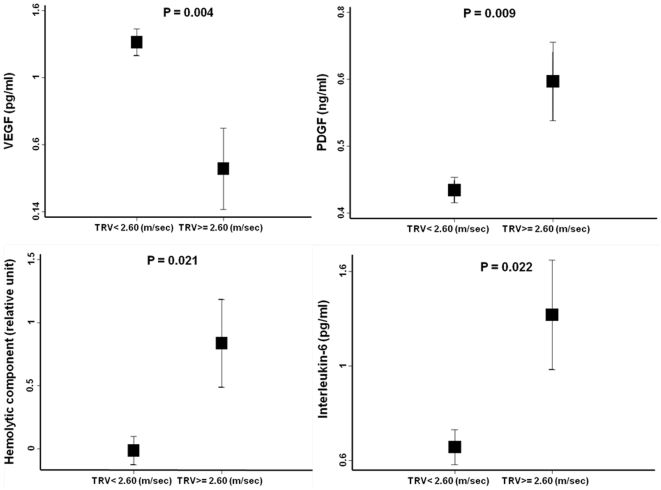
Adjusted mean values VEGF, PDGF-BB, interleukin-6 and hemolytic component according to tricuspid regurgitation velocity category. Presented are least square mean (±standard error) values from ANOVA with adjustment for the other variables depicted. TRV indicates tricuspid regurgitation velocity.

Plasma VEGF concentration had significant positive relationships with PDGF-BB (Figure 1a) and interleukin-6 (Figure 1b) but not with tricuspid regurgitation velocity (Table 3) in bivariate analyses. VEGF had a negative rather than positive independent relationship with tricuspid regurgitation velocity in the multivariate logistic regression analysis (Table 4). Figure 2a shows the mean VEGF concentration was lower in children with tricuspid regurgitation velocity ≥2.60 m/sec than those with velocity <2.60 m/sec after adjustment for PDGF-BB, hemolytic index and interleukin-6. In contrast, Figure 2b–d shows that the adjusted mean values for PDGF, interleukin-6 and hemolytic index were higher with tricuspid regurgitation velocity ≥2.60 m/sec. These observations suggest that the relationship of VEGF with tricuspid regurgitation velocity is modified by the degree of elevation of other angiogenic and inflammatory molecules.

### Pathway Analysis of Tricuspid Regugitation Velocity in Patients with Sickle Cell Disease (Figure 3)

The model had a good fit to the data (Chi-square = 9.1, degrees of freedom = 5, P = 0.11) and it was statistically significant (root mean square error of approximation = 0.05; 90% confidence interval of 0.0 to 0.1). According to this analysis, higher hemolytic component was a direct predictor of increasing tricuspid regurgitation velocity (beta = 0.38) and it also was associated with lower hemoglobin concentration and higher interleukin-6 concentration. Lower hemoglobin concentration was not a direct predictor of higher regurgitation velocity, but it was associated with higher VEGF concentration. Higher interleukin-6 concentration was a direct predictor of increasing tricuspid regurgitation velocity (beta = 0.22) and it also was associated with higher VEGF and PDGF-BB concentrations. Higher PDGF-BB concentration was a direct predictor of increasing regurgitation velocity (beta = 0.25) while higher VEGF concentration was a direct predictor of lower regurgitation velocity. The model predicted 22% of variation in tricuspid regurgitation velocity among patients.

**Figure 3:**
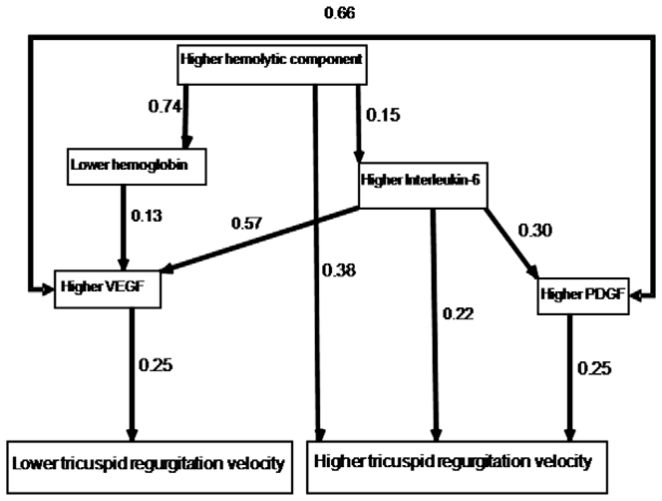
Pathway analysis of tricuspid regurgitation velocity in sickle cell disease patients. The numbers shown are standardized betas. Each of the arrows depicts a significant relationship (P<0.05). According to this analysis, higher hemolytic component was a direct predictor of increasing tricuspid regurgitation velocity and it also was associated with lower hemoglobin concentration and higher interleukin-6 concentration. Lower hemoglobin concentration was not a direct predictor of higher regurgitation velocity, but it was associated with higher VEGF concentration. Higher interleukin-6 concentration was a direct predictor of increasing tricuspid regurgitation velocity and it also was associated with higher VEGF and PDGF-BB concentrations. Higher PDGF-BB concentration was a direct predictor of increasing regurgitation velocity while higher VEGF concentration was a direct predictor of lower regurgitation velocity.

## Discussion

Hemolysis and hypoxia have been identified as risk factors for elevated tricuspid regurgitation velocity in children and adolescents with sickle cell disease [6]. Here we investigated whether markers of other biologic pathways implicated in pulmonary hypertension in conditions other than sickle cell disease may be associated with elevated tricuspid regurgitation velocity in sickle cell disease as well. We also examined the relationship of these markers to mitral valve E/Etdi, an indicator of left ventricular diastolic function. In this process, we found that, in addition to a composite marker of hemolysis, circulating angiogenic and inflammatory markers were associated with elevated tricuspid regurgitation velocity but not with elevated mitral valve E/Etdi ratio in children and adolescents with sickle cell disease. In multivariate analyses, higher levels of PDGF and lower levels of VEGF were associated with elevated regurgitation velocity.

Pulmonary hypertension is characterized by abnormal proliferation of pericytes and smooth muscle cells in the pulmonary microvasculature [38]. PDGF activates vascular smooth muscle cells/pericytes in the process of angiogenesis [39] and promotes the development of experimental and hypoxia-related pulmonary hypertension by inducing the proliferation and migration of smooth muscle cells and fibroblasts [40], [41]. In the present study, we found that greater plasma concentrations of PDGF correlated significantly with higher values for tricuspid regurgitation velocity in both bivariate and multivariate analyses among children and adolescents with sickle cell disease. Other clinical and translational studies also support a role for PDGF in promoting [40], [41] the development of pulmonary hypertension in settings other than sickle cell disease. The pharmacologic agent, imatinib, serves as a PDGF receptor antagonist and reverses vascular remodeling and cor pulmonale in experimental pulmonary hypertension [42]. Recent studies indicate that PDGF receptor antagonists such as imatinib and sunatinib are effective therapy for experimental pulmonary hypertension [42], [43], and case reports suggest that imatinib has efficacy in human patients as well [44]. In addition, activated platelets might be a source of increased plasma PDGF levels in sickle cell disease [22].

In contrast to the stimulation of vascular smooth muscle cells by PDGF, VEGF stimulates proliferation and migration of endothelial cells in the process of angiogenesis [39] and has not consistently been found to contribute to experimental pulmonary hypertension [29], [45], [46]. VEGF expression is controlled by a number of complex regulatory mechanisms [47]. Specifically during hypoxia VEGF transcription is induced from the rapid stabilization of hypoxia inducible factor-1 [48]. During hemolysis and subsequent hypoxia in sickle cell disease, one would expect VEGF to be induced, and the present data shows a strong inverse correlation of circulating VEGF concentration with hemoglobin concentration. Consistent with a recent study in adults [49], we did not observe a significant relationship of plasma concentrations of VEGF with tricuspid regurgitation velocity in bivariate analysis. However, we found that greater VEGF concentrations were associated with lower tricuspid regurgitation velocity in multivariate analysis. This observation is noteworthy in the light of recent research by other investigators indicating that PDGF and VEGF have dichotomous roles in the regulation of vascular smooth muscle cells/pericytes: PDGF mediates the growth of pericytes by a receptor-mediated mechanism in a model of angiogenesis, and increased VEGF prevents this effect of PDGF [39]. Another potential mechanism whereby VEGF may associate with a lower regurgitation velocity is through induction of nitric oxide: VEGF induces nitric oxide synthesis by endothelial nitric oxide synthesis via an Akt-mediated pathway [50]. Other clinical and translational studies also support a role for VEGF in inhibiting [29], [45], [46] the development of pulmonary hypertension in settings other than sickle cell disease. Thus, higher VEGF expression conceivably could protect from elevation of pulmonary artery pressure in sickle cell disease by two pathways, namely inhibition of pulmonary vascular smooth muscle proliferation and increased production of nitric oxide by endothelial cells.

Although we previously observed significant, independent associations of both higher hemolytic index and lower hemoglobin oxygen saturation with elevated tricuspid regurgitation velocity in this cohort of children and adolescents with sickle cell disease [6], the present study indicated that the hemoglobin oxygen saturation was not significantly associated after adjustment for VEGF, PDGF-BB and interleukin-6. Our findings are therefore consistent with the possibility that effects of hypoxia on systolic pulmonary arterial pressure are at least in part mediated by changes in angiogenic and inflammatory responses.

Sickle cell disease is characterized by chronic low-grade inflammation and endothelial activation [51] as manifested by leukocytosis and monocytosis [52], [53] and increased soluble vascular cell adhesion molecules [18], [54], [55]. There are differing reports regarding the involvement of cytokines as mediators of inflammation in sickle cell disease [56], [57], [58], [59]. In the present study, we observed higher plasma levels of interleukin-8 and interleukin-10 in sickle cell disease patients compared to control subjects. Among the patients with sickle cell disease, we observed bivariate associations of higher interleukin-6, interleukin-8, interferon-γ, tumor necrosis factor-α and RANTES concentrations with higher tricuspid regurgitation velocity, and an independent association of increased interleukin-6 concentration with elevated tricuspid regurgitation velocity. These observations are compatible with the possibility that pro-inflammatory processes contribute to pulmonary hypertension in sickle cell disease as proposed by other investigators [21] and as observed in other settings [23], [24], [25], [26], [60]. Our observation that the relationship of VEGF with tricuspid regurgitation velocity is modified by the degree of elevation of the inflammatory marker, interleukin-6, is paralleled by a report from the literature that pulmonary hypertension in tumor necrosis factor-α-over-expressing mice is associated with decreased VEGF expression [61].

There are a number of limitations to our study. Thirty-eight percent of the patients were being treated with hydroxyurea and 13% were on a chronic transfusion program. Although plasma concentration of PDGF was lower in the patients receiving hydroxyurea and higher in those on a chronic transfusion program, these forms of treatment were not significant covariates in examining the relationships of the biologic markers with tricuspid regurgitation velocity or mitral valve E/Etdi. The reliability of single echocardiographic measurements of tricuspid regurgitation velocity and E/Etdi has not been established in children with sickle cell disease. Other than a greater decline in oxygen saturation during the six minute walk test in patients with elevated tricuspid regurgitation velocity, we have not observed functional impairment in patients with elevated tricuspid regurgitation velocity or mitral valve E/Etdi in children and adolescents with sickle cell disease [6]. Other limitations to our study are that circulating concentrations of biologic markers may not reflect the levels to which cells of the pulmonary microvasculature are exposed, that this was a cross-sectional rather than a longitudinal study, and that multiple comparisons dilute the statistical significance of individual observations. Furthermore, the negative association of VEGF with tricuspid regurgitation velocity appears only after adjustment for PDGF concentration. However, the pathway analysis lends support to the finding of contrasting associations of VEGF and PDGF with tricuspid regurgitation velocity.

Although the clinical importance of elevated tricuspid regurgitation velocity in children and adolescents with sickle cell disease has not been clarified by long-term follow-up studies, it seems possible that elevated velocity in this age group may identify individuals at risk for developing pulmonary hypertension later in life. From this standpoint, the PUSH cohort could have unique research importance for studying the development of pulmonary hypertension in sickle cell disease. Our observation of opposing VEGF and PDGF profiles in normal versus elevated tricuspid regurgitation velocity groups supports the idea that elevated velocity associates with known factors involved in pulmonary hypertension, and that this clinical measure may be useful for dissecting the pathogenesis of the early stages of cardiopulmonary complications of sickle cell disease. In general, our findings support the idea that the etiology of pulmonary hypertension in sickle cell disease is multi-factorial, and that pro-inflammatory and angiogenic pathways may interact with the degree of hemolysis in contributing to the development of pulmonary hypertension.
